# INTegrated Assessment of intERmediate Coronary Stenoses by Fractional Flow rEserve and Near-infraREd Spectroscopy: The INTERFERE Study

**DOI:** 10.3390/jcm14196769

**Published:** 2025-09-25

**Authors:** Andrea Picchi, Gianluca Campo, Leonardo Misuraca, Pasquale Baratta, Antonio Biancofiore, Paolo Calabria, Alberto Massoni, Ugo Limbruno

**Affiliations:** 1 Cardiovascular Department, Misericordia Hospital, 58100 Grosseto, Italy; pasquale.baratta@uslsudest.toscana.it (P.B.); antonio.biancofiore@uslsudest.toscana.it (A.B.); paolo.calabria@uslsudest.toscana.it (P.C.); alberto.massoni@uslsudest.toscana.it (A.M.); ugo.limbruno@uslsudest.toscana.it (U.L.); 2 Cardiovascular Institute, Azienda Ospedaliero-Universitaria di Ferrara, 44121 Ferrara, Italy; cmpglc@unife.it; 3 Azienda Ospedaliero-Universitaria Pisana, 56122 Pisa, Italy; leo.misuraca@gmail.com

**Keywords:** fractional flow reserve, near infrared spectroscopy, coronary imaging

## Abstract

**Background/Objectives:** Fractional flow reserve (FFR) is the most widely used intracoronary physiological index to guide coronary revascularization but does not allow for a precise assessment of plaque morphology. The combined use of near-infrared spectroscopy (NIRS) and intravascular ultrasound (IVUS) can detect angiographically non-obstructive lesions with high lipid content and large plaque burden, which are associated with an increased risk of future adverse cardiac events. The aim of this study is to perform an integrated assessment of angiographically intermediate coronary lesions using both FFR and IVUS-NIRS, in order to evaluate the distribution of plaque vulnerability features—assessed by IVUS-NIRS—in functionally significant and non-significant lesions. **Methods:** This was a double-center, observational, prospective study including patients undergoing coronary angiography for both stable coronary artery disease and acute coronary syndrome, provided they had at least one angiographically borderline (40–70%) stenosis. The index lesion was evaluated with both FFR and IVUS-NIRS; revascularization decisions were guided by the FFR result. The following features were considered markers of plaque vulnerability: minimal lumen area (MLA) < 4.0 mm^2^, plaque burden (PB) > 70%, and maximum lipid core burden index within any 4 mm segment (maxLCBI4mm) > 325. High-risk plaques were defined by the simultaneous presence of all three criteria. **Results:** A total of 57 patients were enrolled (mean age: 66 years; 18% women), and 57 lesions were assessed using both FFR and IVUS-NIRS. Acute coronary syndrome was the admission diagnosis in 72% of patients. Twenty-five lesions with FFR < 0.80 were classified as Group A, while the remaining thirty-two lesions with FFR > 0.80 were labeled as Group B. The percentage of lesions with MLA < 4 mm^2^ and plaque burden > 70% was 72% and 67%, respectively, with no significant differences between Groups A and B. On NIRS analysis, 23% of lesions had a maxLCBI4mm > 325, again with no significant difference between the two groups. High-risk plaques—defined by the concurrent presence of MLA < 4 mm^2^, plaque burden > 70%, and maxLCBI4mm > 325—were identified in 18% of patients. The prevalence of high-risk plaques did not differ significantly between Groups A and B (12% vs. 22%, *p* = 0.33). **Conclusions:** Plaque vulnerability criteria are equally distributed between functionally significant and non-significant coronary lesions, and the prevalence of high-risk plaques (defined by the simultaneous presence of MLA < 4 mm^2^, PB > 70%, and maxLCBI4mm > 325) does not differ significantly between the two groups. Notably, 22% of FFR-negative lesions managed conservatively are characterized by the presence of high-risk plaques. Further studies are needed to determine whether these lesions warrant interventional treatment or a more intensive pharmacological approach. (ClinicalTrials.gov # NCT02985112).

## 1. Introduction

Fractional flow reserve (FFR) is the most widely used intracoronary physiology index to guide coronary revascularization strategy in the catheterization laboratory. The safety of FFR as a decision-making tool has been documented in studies that mainly involved patients affected by stable coronary artery disease [1] but has recently also been demonstrated in the context of acute coronary syndromes [2,3].

Multiple large, randomized trials showed that stenoses with an FFR < 0.80 have a poor prognosis and require treatment by PCI whereas PCI of lesions with FFR > 0.80 can be safely deferred [4]. The safety of deferring revascularization in FFR-negative lesions stems from the benefit of identifying lesions that are non-flow limiting and with a low risk of triggering acute ischemic events. However, FFR does not allow precise assessment of plaque morphology and cannot detect atherosclerotic lesions with unstable features, which may identify patients at higher risk of acute coronary events while presenting without flow limitation [5].

In previous intravascular ultrasound (IVUS) studies, lesion characteristics that were predictive of events associated with non-culprit lesions included a large plaque burden, a small luminal area, and thin-cap fibroatheromas (TFCA) [6]. In diabetic patients mainly affected by stable coronary artery disease, optical coherence tomography (OCT) allowed the detection of unstable plaques with TFCA in 25% of FFR-negative lesions, which were associated with a five-fold higher rate of MACE despite the absence of ischemia [7]. Among patients with myocardial infarction and FFR-negative non-culprit lesions, the presence of a high-risk plaque, defined by OCT analysis, was associated with a worse clinical outcome [8].

Near-infrared spectroscopy is one imaging modality that allows identification of vulnerable plaques, using automated detection and quantification of lipid content [9]. In the PROSPECT II study, the combined use of NIRS and intravascular ultrasound allowed us to detect angiographically non-obstructive lesions with a high lipid content and large plaque burden that were at increased risk for future adverse cardiac outcomes [10].

Similarly, the Lipid Rich Plaque (LRP) study investigated the relationship between the presence of lipid-rich plaque detected by near-infrared spectroscopy (NIRS) and intravascular ultrasound (IVUS) imaging at unstented sites and the occurrence of subsequent major adverse cardiac events (MACE): this study showed that the presence of a high maxLCBI4mm (maximum Lipid Core Burden Index within a 4 mm segment) was an independent predictor of future non-culprit events [11].

In the PREVENT trial, treatment of vulnerable plaques, detected by OCT and IVUS-NIRS criteria, with a preventive percutaneous coronary intervention strategy reduced the risk of major cardiovascular events compared to medical therapy [12].

The aim of this study is to perform an integrated assessment of angiographically intermediate coronary lesions by FFR and IVUS-NIRS and to evaluate the distribution of plaque vulnerability features, assessed by IVUS-NIRS, in functionally significant and non-significant lesions.

## 2. Material and Methods

The INTERFERE (NCT02985112) is an observational, prospective study that was conducted in two Italian sites (Misericordia Hospital, Grosseto and University of Ferrara, Ferrara).

Subjects undergoing coronary angiography for stable coronary artery disease, non-ST-segment elevation acute myocardial infarction (NSTEMI), and unstable angina were eligible for enrollment if they had at least one angiographically borderline stenosis (≥40, <70% by Quantitative Coronary Angiography, QCA) with normal antegrade flow (TIMI 3). The index lesion was first evaluated by FFR; subsequently, plaque composition and lesion characteristics were assessed using intravascular ultrasound (IVUS) and NIRS. Revascularization of the index lesion was performed after completing the functional and morphological assessment and was guided by FFR findings in accordance with current guidelines on myocardial revascularization [13]: patients with exclusively FFR-positive lesions (i.e., FFR < 0.80) underwent mandatory revascularization. Patients with FFR-negative index lesions (i.e., FFR > 0.80) were treated by guideline-recommended optimal medical therapy.

Patients with hemodynamic instability, ST-segment-elevation myocardial infarction, known allergy to antiplatelet or anticoagulant drugs, history of previous surgical revascularization, significant left main disease, life expectancy <1 year, severe renal failure, malignancy, scheduled valve surgery, inability to provide informed consent, known bronchial asthma, or age < 18 were excluded.

FFR was measured with an intracoronary pressure guidewire (Radi pressure wire 4; Radi Medical Systems). An intravenous infusion of adenosine (Adenoscan; Sanofi Aventis, Milan, Italy) at 140 µg/kg/min was then administered to induce a steady-state maximal hyperemia. Simultaneous measurements of mean aortic pressure (through the guiding catheter) and mean distal coronary pressure (by the pressure wire) were obtained in the resting and maximal hyperemic states. FFR was then calculated as the ratio of mean distal coronary pressure to mean aortic pressure at maximal hyperemia.

The IVUS-NIRS system, as used in this study, consists of a 3.2-F rapid exchange catheter, a pullback and rotation device, and a console (InfraReDx, Burlington, MA, USA). Image acquisition was performed by a motorized catheter pullback at a speed of 0.5 mm/s and 240 rpm in the target vessel, starting distal to the index lesion. The system performed 1000 chemical measurements per 12.5 mm, in which each measurement interrogated 1 to 2 mm^2^ of vessel wall from a depth of approximately 1 mm in the direction from the luminal surface toward the adventitia [9]. NIRS data generated a chemogram, which is a color-coded distribution of lipid probability with the x-axis corresponding to the axial vessel position (0·1 mm per pixel) and the y-axis corresponding to the circumferential position (1° per pixel). Low probability of lipids is shown as red and high probability of lipids is shown as yellow [14]. The quantitative expression of NIRS results is referred to as the Lipid Core Burden Index (LCBI), a dimensionless numerical representation of the chemogram. The LCBI, as assessed by NIRS, is calculated as the fraction of pixels with a lipid probability > 0.6 divided by the total number of analyzable pixels within the interrogated vessel segment, multiplied by 1000. The resulting value ranges from 0 to 1000, corresponding to a 0–100% lipid content. The maxLCBI4mm is defined as the maximum LCBI value found within any 4 mm segment of the entire lesion.

In line with prior exploratory findings from the PROSPECT and PROSPECT II studies, the following features were considered indicative of vulnerable plaque: minimal lumen area < 4.0 mm^2^, plaque burden > 70% by IVUS, and maxLCBI4mm > 325 by NIRS. A high-risk plaque phenotype was defined as the concurrent presence of all three features.

Imaging data were analyzed offline by an independent, blinded core laboratory (Euroimaging Srl, Rome, Italy).

The occurrence of major adverse cardiovascular events (MACEs)—defined as a composite of all-cause mortality, non-fatal myocardial infarction, or unplanned revascularization—was assessed during long-term follow-up. Given the observational design of the study, the evaluation of MACEs and plaque vulnerability features were exploratory in nature, aimed at generating hypotheses for future research rather than testing predefined clinical endpoints.

### Statistical Analysis

The normal distribution of continuous variables was assessed using the Kolmogorov–Smirnov test. Continuous variables are presented as mean (standard deviation [SD]) or median (interquartile range [IQR]), as appropriate. Comparisons between groups were performed using Student’s *t*-test for normally distributed variables or the Mann–Whitney *U* test for non-normally distributed variables. Categorical variables are expressed as counts and percentages and were compared using the Pearson chi-square test or Fisher’s exact test, where appropriate. A two-sided *p*-value ≤ 0.05 was considered statistically significant.

The sample size was estimated to explore a potential reduction in the prevalence of plaques with a maxLCBI4mm > 400 from 36% in FFR-positive lesions to 18% in FFR-negative lesions. This estimate was based on prior observations showing a 36% vs. 18% prevalence of thin-cap fibroatheromas in lesions with >70% vs. <70% diameter stenosis. Using a chi-square test for 2 × 2 tables and a one-sided alpha level of 0.05, a sample of 150 lesions was calculated to provide 80% power to detect the expected difference. To describe differences in lipid core burden across the index lesion, between-group effect sizes were calculated using Cohen’s and categorized as small (d ≥ 0.20 and <0.50), medium (d ≥ 0.50 and <0.80), or large (d ≥ 0.80). A *p*-value < 0.05 was considered indicative of statistical significance.

The analysis of major adverse cardiovascular events (MACEs) was conducted by comparing patients with and without high-risk plaques, defined by the presence of specific imaging features. Patients were censored at their last available follow-up.

To assess the independent association between high-risk lesions and the occurrence of the composite MACE endpoint, we fitted a multivariable logistic regression model including clinically pre-specified baseline covariates (age, sex, diabetes, hypertension, hypercholesterolemia, smoking, and serum creatinine). Because of the limited sample size and the presence of quasi-separation in the data, Firth’s penalized likelihood approach was applied to reduce small-sample bias and obtain finite and unbiased maximum likelihood estimates. Odds ratios (ORs) with corresponding 95% confidence intervals (CIs) and Wald *p*-values were reported for the exposure of interest and all covariates.

All statistical analyses were performed using Prism software 8.0 (GraphPad Software, 225 Franklin Street, Fl. 26, Boston, MA, USA).

## 3. Results

From March 2015 to December 2019, a total of 57 patients in two Italian study centers were enrolled in the study and 57 lesions were analyzed by both FFR and IVUS-NIRS.

Table 1 shows patient demographics and procedural characteristics. Median age was 66 years and 18% of the patients were female. The majority of patients (>70%) were treated for acute coronary syndromes and only 28% of them was admitted with a diagnosis of stable coronary disease. A quarter of patients had a previous acute coronary syndrome and a third of them a previous percutaneous coronary revascularization (Table 2).

Most patients were discharged with an indication to dual antiplatelet therapy and optimized lipid-lowering therapy (i.e., maximum tolerated dose) throughout the follow-up period. The index lesion was located in the LAD in almost 70% of cases.

Twenty-five lesions showed a FFR value < 0.80, were treated by PCI and represented Group A: the remaining 32 lesions with a negative FFR (>0.80) and addressed to medical treatment were labeled as Group B.

**Figure 1:**
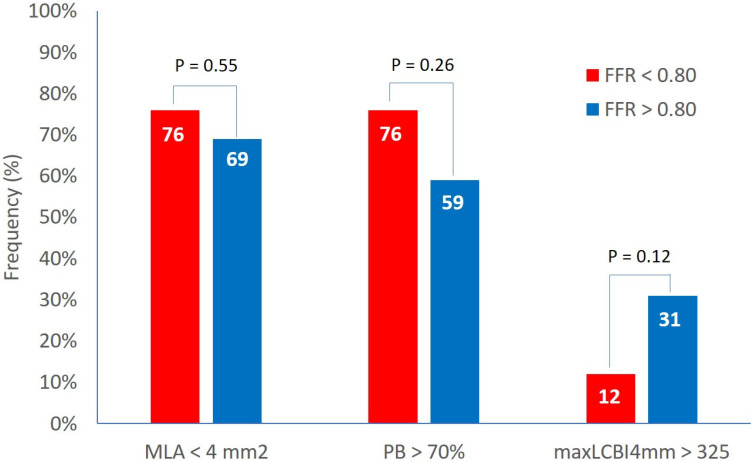
Prevalence of high-risk features in functionally significant and non-significant lesions. FFR = fractional flow reserve, MaxLCBI4mm = maximum lipid core burden index within any 4 mm segment, MLA = minimal lumen area, PB = plaque burden.

Clinical and demographics characteristics were similar in both groups. The IVUS analysis showed an MLA of 3.6 ± 1.2 mm^2^ and a plaque burden at the site of MLA of 73.3 ± 7.9% with no difference between the two groups. The percentage of lesions with MLA < 4 mm^2^ and with plaque burden > 70% was 72% and 67%, respectively, with non-significant differences between Groups A and B (Figure 1).

At the NIRS analysis, the LCBI 4 mm around MLA was 204 ± 160 and the percentage of patients with an LCBI 4 mm around MLA > 325 was 23% with no difference between the two groups and no effect size (effect size Cohen’s *d*: 0.1) (Figure 1).

**Figure 2:**
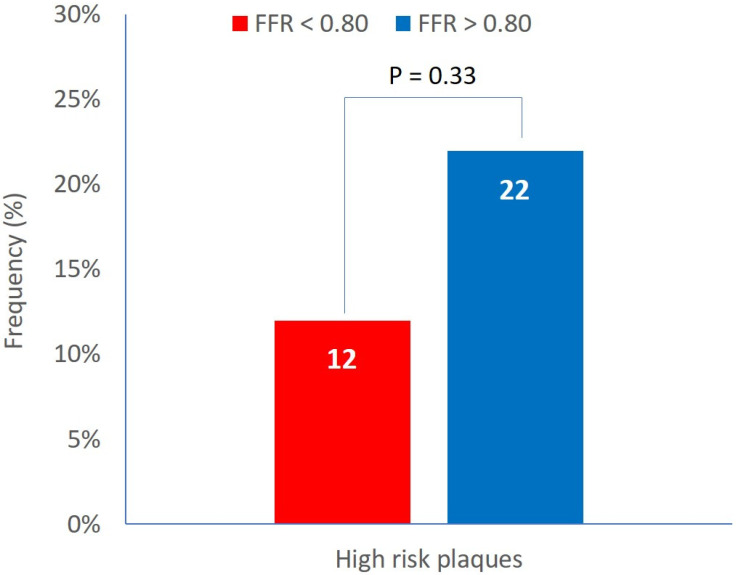
Prevalence of high-risk plaques in functionally significant and non-significant lesions.

High-risk plaques (defined by the simultaneous presence of the three plaque vulnerability features, MLA < 4 mm^2^, plaque burden > 70%, and LCBI 4 mm around MLA > 325) were detected in the 18% of patients. The prevalence of high-risk plaques was not different between Groups A and B (12% vs. 22%, *p* = 0.33) (Figure 2).

The incidence of MACEs (a composite of cardiovascular death, myocardial infarction, and unplanned revascularization) was also assessed at a mean follow-up of 2896 ± 441 days in subjects with both high-risk and non-high-risk lesions. In the overall population, only one myocardial infarction and two unplanned revascularizations occurred during long-term follow-up, all of which were related to the index lesion.

In the multivariable Firth logistic regression model, the presence of high-risk lesions was independently associated with a higher risk of the composite MACE endpoint. Among baseline covariates, none showed a statistically significant independent effect after adjustment. Odds ratios with 95% confidence intervals and corresponding *p*-values are reported in Table 3.

**Figure 3:**
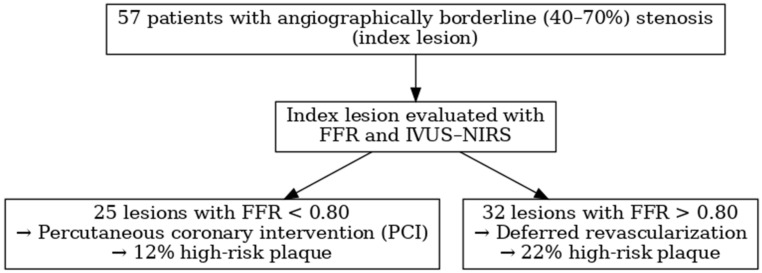
STROBE flow diagram.

A STROBE flow diagram summarizing the study design and results is presented in Figure 3.

## 4. Discussion

The major findings of this study are as follows: (1) plaque vulnerability criteria are equally distributed between functionally significant and non-significant coronary lesions, (2) the prevalence of high-risk plaques—defined by the simultaneous presence of three vulnerability features (MLA < 4 mm^2^, PB > 70%, and maxLCBI_4_mm > 325)—does not differ significantly between functionally significant and non-significant lesions, (3) twenty-two percent of FFR-negative lesions, managed with pharmacological treatment, are characterized by the presence of high-risk plaques.

Our study provides new insights into the relationship between plaque composition and FFR values, clearly demonstrating that non-flow-limiting lesions can exhibit morphological features of plaque vulnerability, similar to those observed in functionally significant stenoses, which are typically treated invasively.

Similar results were observed in the COMBINE OCT–FFR trial [7]. In that study, OCT-detected vulnerable plaques were present in up to 25% of angiographically intermediate, FFR-negative lesions and were responsible for over 80% of future adverse events despite optimal medical therapy. Conversely, the remaining 75% of FFR-negative lesions without vulnerability features were associated with a low risk of future events. The percentage of high-risk, non-significant stenoses in the COMBINE OCT–FFR trial (25%) closely aligns with our findings (22%). The slight discrepancy may be explained by differences in study populations: the COMBINE OCT–FFR cohort consisted exclusively of diabetic patients.

A higher prevalence of high-risk plaques was reported in the PECTUS trial (34%), which enrolled only patients with acute coronary syndromes (STEMI and NSTEMI) [8]. In such populations, the detection of vulnerable plaques in non-culprit lesions is expected to be more frequent.

In the more recent PREVENT trial, the prevalence of high-risk plaques among FFR-negative lesions was substantially higher (45%) [12]. This may be attributed to the use of less stringent criteria for plaque vulnerability, defined by the presence of only two features. Additionally, the adoption of a lower maxLCBI cutoff (315) may have contributed to the higher observed prevalence.

**Figure 4:**
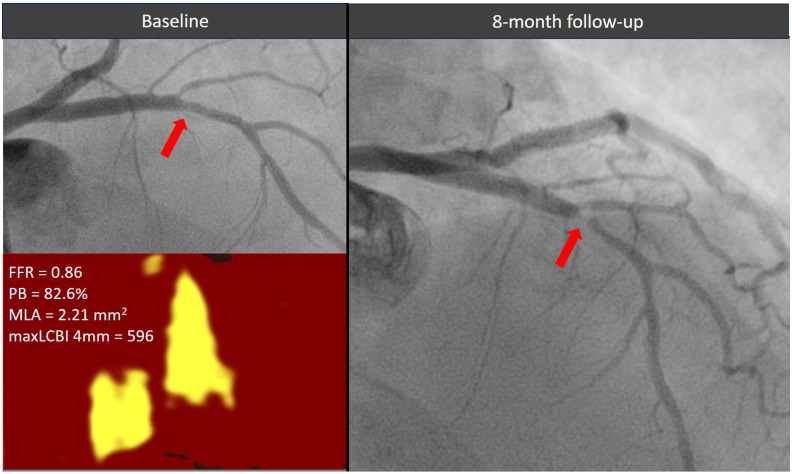
Evolution of a functionally non-significant proximal left descending artery lesion (red arrow) exhibiting two high-risk morphological features. The plaque showed a significant stenosis progression causing an acute coronary syndrome within 1 year from the index angiogram. FFR = fractional flow reserve, MaxLCBI4mm = maximum lipid core burden index within any 4 mm segment, MLA = minimal lumen area, PB = plaque burden.

The optimal treatment strategy for functionally non-significant lesions with high-risk morphological features remains a topic of ongoing debate. Figure 4 shows a case of a functionally non-critical coronary lesion with high-risk morphological features, initially managed with medical therapy, which subsequently led to a coronary event within a short time requiring revascularization. In the PREVENT study, prophylactic percutaneous coronary intervention (PCI) of non-flow-limiting, vulnerable plaques was associated with a lower incidence of major adverse cardiac events during long-term follow-up compared with pharmacological therapy [12]. However, medical therapy in the study was largely suboptimal, with limited use of PCSK9 inhibitors and relatively high LDL-cholesterol targets (60 to 85 mg/dL). Previous studies have demonstrated that adding PCSK9 inhibitors to high-intensity statin therapy in patients with acute myocardial infarction leads to favorable effects on plaque regression and stabilization, including reductions in lipid burden and increases in fibrous cap thickness [15,16].

Notably, the population with FFR > 0.80 includes a lower proportion of individuals with hypertension and those who smoke. Hypertension and smoking individually elevate cardiovascular risk and together they exert compounded adverse effects on coronary plaque formation and progression. Hypertension contributes via endothelial injury, increased arterial wall stress, and promotion of calcification, while smoking accelerates atherosclerosis through oxidative stress, inflammation, lipid dysfunction, and prothrombotic activity [17]. The Heinz Nixdorf Recall study showed a graded increase in coronary artery calcium progression with increasing blood pressure categories [18]. Smokers had a significantly higher prevalence and volume of coronary plaques including lipid, fibrous, and calcified types, independent of other risk factors [19]. Smokers were also more likely to harbor lipid-rich, vulnerable plaques assessed by intravascular ultrasound which are prone to rupture [20]. Although the plaque burden at the index lesion does not differ between FFR-positive and FFR-negative lesions, it could be argued that patients with functionally non-significant lesions—and a lower prevalence of smoking and hypertension—may have less diffuse coronary artery disease and no stress-induced myocardial ischemia.

Based on current evidence, the actual impact of functionally non-significant lesions with high-risk morphological features on cardiovascular outcomes remains uncertain and the optimal treatment strategy for such lesions should be evaluated in large-scale randomized trials.

It is worth noting that non-invasive imaging modalities are now available to assess both plaque morphology and the functional significance of coronary stenoses. Coronary computed tomography angiography (CCTA) has emerged as a precise tool to detect, quantify, and characterize coronary atherosclerotic plaque [21,22]. Several imaging features are associated with plaque vulnerability, including low-attenuation areas, positive remodeling, spotty calcifications, the napkin–ring sign, and increased perivascular fat attenuation index (FAI), all of which have demonstrated prognostic value. By identifying these high-risk features, CCTA enhances risk prediction for major adverse cardiovascular events (MACE), particularly in patients with non-obstructive disease, as well as in younger individuals and women. In addition, non-invasive fractional flow reserve (FFR) can be derived from standard CCTA acquisitions, providing high diagnostic accuracy and excellent concordance with invasive FFR for identifying hemodynamically significant coronary artery disease [23]. A combined non-invasive functional and morphological assessment of CAD is therefore likely to become the gold standard diagnostic approach in most patients with suspected coronary artery disease in the near future.

### Limitations

This study has several limitations. First, the small sample size may have introduced bias, as the final number of lesions included was lower than initially anticipated based on the sample size calculation. This was primarily due to a low enrollment rate and the discontinuation of IVUS-NIRS availability at our center.

Second, IVUS-NIRS analysis has not been performed in all three coronary vessels; therefore, we might have missed high-risk lesions. However, such targeted evaluation might be more feasible in clinical practice. Third, we did not perform FFR pullbacks, which may further help distinguish between focal and diffuse disease patterns. Fourth, operators were not blinded to IVUS-NIRS findings, which could potentially have led to altered decision-making. However, the IVUS-NIRS analyses were performed post hoc by an independent core laboratory.

## 5. Conclusions

In this population predominantly affected by acute coronary syndrome, plaque vulnerability features were observed to be similarly distributed between functionally significant and non-significant coronary lesions, with no statistically significant difference in the prevalence of high-risk plaques between these groups. Notably, a substantial proportion—over one-fifth—of FFR-negative lesions managed conservatively exhibited high-risk characteristics typically associated with increased risk of adverse events. These observations suggest that relying solely on functional assessment may overlook a subset of lesions with potentially unstable plaque morphology. However, the clinical implications of these findings remain uncertain, as it is not yet clear whether patients with FFR-negative but morphologically high-risk plaques would derive benefit from interventional therapies or from more aggressive pharmacological strategies. Therefore, prospective studies with long-term follow-up are warranted to better define the prognostic significance of these lesions and to guide personalized management strategies aimed at reducing future coronary events.

## Figures and Tables

**Table 1:** Baseline characteristics.

	Total	FFR < 0.80	FFR > 0.80	*p*-Value
	**N = 57**	**N = 25**	**N = 32**	
Age	65.7 ± 10	67.1 ± 11	64.7 ± 10	0.51
Male_gender N (%)	47 (82)	22 (88)	25 (78)	0.33
Diabetes	16 (28%)	8 (32%)	8 (25%)	0.57
Previous ACS	14 (25%)	9 (36%)	5 (16%)	0.12
Previous PCI	19 (33%)	11 (44%)	8 (25%)	0.16
Previous stroke	3 (5%)	0 (0%)	3 (9%)	0.25
PAD	6 (11%)	4 (16%)	2 (6%)	0.39
Presentation				
STEMI	5 (9%)	1 (4%)	4 (12%)	0.37
NSTEMI	21 (37%)	10 (40%)	11 (34%)	0.78
Unstable angina	15 (26%)	7 (28%)	8 (25%)	0.99
Stable CAD	16 (28%)	7 (28%)	9 (28%)	0.99
BMI (kg/m^2^)	25.5 ± 6.3	24.9 ± 6.7	26.1 ± 5.9	0.48
Hypertension	41 (72%)	23 (92%)	18 (56%)	0.003
Hypercholesterolemia	34 (60%)	18 (72%)	16 (50%)	0.11
Family history of CAD	12 (21%)	4 (16%)	8 (25%)	0.52
Active smoker	12 (21%)	9 (36%)	3 (9%)	0.02
Past smoker	20 (35%)	8 (32%)	12 (37%)	0.78
Diabetes	16 (28%)	8 (32%)	8 (25%)	0.57
ASA	55 (96%)	25 (100%)	30 (94%)	0.50
Clopidogrel	18 (32%)	11 (44%)	7 (22%)	0.09
Prasugrel	1 (2%)	0 (0%)	1 (3%)	0.99
Ticagrelor	27 (47%)	12 (48%)	15 (47%)	0.99
Betablocker	42 (74%)	18 (72%)	24 (75%)	0.99
ACEI or ARB	52 (91%)	23 (92%)	29 (91%)	0.99
Statin	54 (95%)	23 (92%)	31 (97%)	0.58
Total cholesterol (mg/dL)	170.8 ± 49.7	173.1 ± 50.1	169.8 ± 48.5	0.83
LDL cholesterol(mg/dL)	98.8 ± 46.7	97.3 ± 48.2	100 ± 46.6	0.86
HDL cholesterol (mg/dL)	45.1 ± 11.3	44.6 ± 10.2	45.4 ± 12.3	0.82
Triglyceride (mg/dL)	136.3 ± 85.4	158.5 ± 110.9	119.2 ± 55.8	0.16
Creatinine (mg/dL)	1.09 ± 0.50	1.04 ± 0.4	1.13 ± 0.57	0.55
GFR (mL/min)	79.9 ± 29.3	80.1 ± 33.3	79.0 ± 26.0	0.83
Number of diseased vessels				
1	24 (42%)	6 (24%)	18 (56%)	0.02
2	21 (37%)	11 (44%)	10 (31%)	0.41
3	12 (21%)	8 (32%)	4 (12%)	0.10
SYNTAX score	12.0 ± 5.4	13.8 ± 5.6	10.6 ± 4.8	0.03
Index lesion LAD	39 (68%)	20 (80%)	19 (59%)	0.15
Index lesion Cx	7 (12%)	5 (20%)	2 (6%)	0.22
Index lesion RCA	11 (19%)	1 (4%)	10 (31%)	0.02
Index lesion MLA (mm^2^)	3.6 ± 1.2	3.6 ± 1.1	3.6 ± 1.2	0.93
Plaque burden at MLA (%)	73.3 ± 7.9	74.2 ± 7.2	72.6 ± 8.4	0.44
LCBImax 4mm around index lesion	204 ± 160	195.2 ± 140.9	210.9 ± 175.3	0.72
MLA < 4 mm^2^	41 (72%)	19 (76%)	22(69%)	0.55
Plaque burden at MLA > 70%	38 (67%)	19 (76%)	19 (59%)	0.26
LCBImax 4 mm around index lesion > 325	13 (23%)	3 (12%)	10 (31%)	0.12
High-risk plaques (MLA < 4 mm^2^ + PB > 70% + LCBImax 4 mm around index lesion > 325)	10 (18%)	3 (12%)	7 (22%)	0.33

ACEI = angiotensin converting enzyme inhibitor, ACS = acute coronary syndrome, ARB = angiotensin receptor blocker, CAD = coronary artery disease, Cx = circumflex coronary artery, GFR = glomerular filtration rate, LAD= left anterior descending coronary artery, LCBI = lipid core burden index, MLA = minimal lumen area, RCA = right coronary artery, PAD = peripheral artery disease, PCI = percutaneous coronary intervention.

**Table 2:** Clinical outcome at long-term follow-up. Major cardiovascular events in patients with and without high-risk lesions: general population. Major cardiovascular events in patients with and without high-risk lesions: subgroup of patients with negative FFR.

	General Population	Patients with High-Risk Lesions	Patients Without High-Risk Lesions	*p*-Value
	**N = 57**	**N = 10**	**N = 47**	
All-cause death	10 (18%)	2 (20%)	8 (17%)	0.82
Myocardial infarction	1 (2%)	0 (0%)	1 (2%)	0.64
Unplanned revascularization	2 (4%)	0 (0%)	2 (4%)	0.51
Composite endpoint: all-cause death, myocardial infarction, unplanned revascularization	12 (21%)	2 (20%)	10 (21%)	0.93
	**General Population**	**Patients with High-Risk Lesions**	**Patients Without High-Risk Lesions**	***p*-Value**
	**N = 32**	**N = 7**	**N = 25**	
All-cause death	6 (19%)	2 (29%)	4 (16%)	0.45
Myocardial infarction	0 (0%)	0 (0%)	0 (0%)	
Unplanned revascularization	1 (3%)	0 (0%)	1 (4%)	0.59
Composite endpoint: all-cause death, myocardial infarction, unplanned revascularization	7 (22%)	2 (29%)	5 (20%)	0.63

**Table 3:** Multivariable Firth logistic regression for MACE composite endpoint.

Predictor	OR (95% CI)	*p*-Value
Age	1.05 (0.98–1.12)	0.135
Hypercholesterolemia	0.38 (0.10–1.47)	0.161
Creatinine (mg/dL)	1.83 (0.41–8.16)	0.425
Male gender	1.76 (0.36–8.63)	0.487
Active smoker	0.69 (0.16–2.96)	0.617
Hypertension	1.41 (0.34–5.84)	0.638
Diabetes	0.72 (0.17–3.06)	0.657
High-risk lesions	0.89 (0.18–4.42)	0.889

Abbreviations: OR, odds ratio; CI, confidence interval; MACE, major adverse cardiovascular events. Model adjusted for pre-specified baseline covariates (age, sex, diabetes, hypertension, hypercholesterolemia, smoking, creatinine). Firth’s penalized likelihood was used to account for small sample size and separation.

## Data Availability

The original contributions presented in this study are included in the article. Further inquiries can be directed to the corresponding author(s).
